# Collagen Type XI Alpha 1 Expression in Intraductal Papillomas Predicts Malignant Recurrence

**DOI:** 10.1155/2015/812027

**Published:** 2015-09-13

**Authors:** Javier Freire, Lucia García-Berbel, Pilar García-Berbel, Saray Pereda, Ainara Azueta, Pilar García-Arranz, Ana De Juan, Alfonso Vega, Ángela Hens, Ana Enguita, Pedro Muñoz-Cacho, Javier Gómez-Román

**Affiliations:** ^1^Anatomía Patológica, Hospital Universitario Marqués de Valdecilla, 39008 Santander, Spain; ^2^IDIVAL, 39011 Santander, Spain; ^3^Ginecología y Obstetricia, Hospital Universitario de Puerto Real, 11510 Puerto Real, Spain; ^4^Oncología Médica, Hospital Universitario Marqués de Valdecilla, 39008 Santander, Spain; ^5^Radiodiagnóstico, Hospital Universitario Marqués de Valdecilla, 39008 Santander, Spain; ^6^Anatomía Patológica, Hospital Universitario de Puerto Real, 11510 Puerto Real, Spain; ^7^Anatomía Patológica, Hospital Universitario 12 de Octubre, 28041 Madrid, Spain; ^8^Gerencia Atención Primaria, Servicio Cántabro de Salud, 39011 Santander, Spain

## Abstract

Despite the progress achieved in the treatment of breast cancer, there are still many unsolved clinical issues, being the diagnosis, prognosis, and treatment of papillary diseases, one of the highest challenges. Because of its unpredictable clinical behavior, treatment of intraductal papilloma has generated a great controversy. Even though considered as a benign lesion, it presents high rate of malignant recurrence. This is the reason why there are clinicians supporting a complete excision of the lesion, while others support an only expectant follow-up. Previous results of our group suggested that procollagen 11 alpha 1 (pro-COL11A1) expression correlates with infiltrating phenotype in breast lesions. We analyzed the correlation between expression of pro-COL11A1 in intraductal papilloma and their risk of malignant recurrence. Immunohistochemistry of pro-COL11A1 was performed in 62 samples of intraductal papilloma. Ten out 11 cases relapsed as carcinoma presents positive staining for COL11A1, while just 17 out of 51 cases with benign behaviour present immunostaining. There were significant differences (*P* < 0.0001) when comparing patients with malignant recurrence versus nonmalignant relapse patients. These data suggest that pro-COL11A1 expression is a highly sensitive biomarker to predict malignant relapse of intraductal papilloma and it can be used as indicative factor for prevention programs.

## 1. Background

Breast cancer is the first tumor disease among women, causing more than 600000 new cases per year [1]. Furthermore it is also the second cause of cancer death among women, causing more than 39,000 deaths each year only in United States [2]. Although in the last years the early detection of this disease has improved overall survival [3], breast cancer remains a very serious problem for public health and there are still open many research areas.

Papillary lesions (intraductal papilloma, papillomatosis, atypical papilloma, and intraductal papillary carcinoma) are controversial and continuously generate problems in diagnosis and clinical management [4]. Because of their similarity, the accurate diagnosis of these lesions only by morphology may be complex, so pathologist requires the use of ancillary techniques. The main indicator of malignancy of papillary lesion is the absence of myoepithelial cells [5] which can be revealed by immunohistochemistry for p63 protein, smooth muscle actin (SMM-HC), or calponin [6]. Other biomarkers have been used as estrogen receptor or cytokeratins [6] CK5/6 and CK8 [7] for differential diagnosis but there is a no clear consensus to determine the sensitivity and accuracy of these markers in routine [5, 8].

Intraductal papilloma is the most controversial papillary lesion relating diagnosis and treatment [6]. While intraductal papilloma* per se* behaves like a benign lesion, the association between intraductal papilloma and malignant recurrence is fairly high, reaching up to 33% of the cases [5, 9, 10]. Indeed, there is a great controversy on how to act when a new case of intraductal papilloma is diagnosed. In fact there are papers suggesting a radical excision of the lesion in all cases [11, 12], while others support only an expectant follow-up [13–15]. An accurate diagnosis pointing to cases amenable of a malignant behavior is essential [6, 16, 17], not only for the benefit of the patient, as it would avoid unnecessary interventions, but also because of its economic impact [8].

It has been shown that the extracellular matrix plays an essential role in breast tumor development and progression, being collagens its main component. Collagen type XI alpha 1 (COL11A1) has been shown to be a marker of malignancy in different tumors including pancreas [18], lung [19], stomach [20], and colon [21–23]. Previous work from our group has demonstrated that pro-COL11A1 expression in cancer associated fibroblasts is a powerful marker of invasive growth in breast carcinomas, with sensitivity and specificity rates higher than 90% [24]. COL11A1 is not present in benign lesions so we thought it can be a predictable marker for malign behavior of intraductal papilloma.

## 2. Mat and Meth

### 2.1. Tissue Samples

Sixty-two patients with a clinicopathological diagnosis of breast intraductal papilloma from the University Hospital Marqués de Valdecilla (Santander, Spain), University Hospital of Puerto Real (Puerto Real, Spain) and University Hospital 12 de Octubre (Madrid, Spain) were enrolled for this work. All the samples examined were core needle biopsies from 18G gauge.

Patients were diagnosed by two independent pathologists following the standard work routine. All patients recruited for the study had a minimum follow-up of 5 years. Patient recruitment was conducted under approvement by the Clinical Research Ethics Committee of Cantabria.

Five cases of encapsulated papillary carcinoma were also selected as positive control of malignant lesion.

### 2.2. Immunohistochemical Analysis

Formalin fixed, paraffin embedded biopsies were stained by using proCol11a1 monoclonal antibody clone 1E8.33 (ONCOMATRYX, Bilbao, SPAIN) as previously described [24, 25]. Samples were considered as positive when a clear cytoplasmic labeling of at least one tumor-associated fibroblast was observed. Staining was separately evaluated by two independent pathologists.

### 2.3. Statistical Methods

Nonparametric Fisher exact test was performed, using SPSS 20 suite, to analyze difference of COL11A1 expression between intraductal papilloma with or without malignant relapse. Survival analyses were performed using Kaplan-Meier curves, and hazard ratio (HR) and corresponding 95% confident interval (95% CI) were estimated using Cox proportional hazards regression of recurrence for positive staining for COL11A1.

## 3. Results

Out of 62 cases studied, 11 presented recurrence as an infiltrative carcinoma, 7 presented further nonmalignant proliferative lesion (papilloma, columnar hyperplasia…) while 44 remaining cases showed no recurrence. Benign relapsed or no recurrence samples were considered as a single group for comparing with those which presented as a malignant relapse. Immunolabeling of pro-COL11A1 was observed in fibroblasts surrounding central fibrovascular stalks.

**Figure 1:**
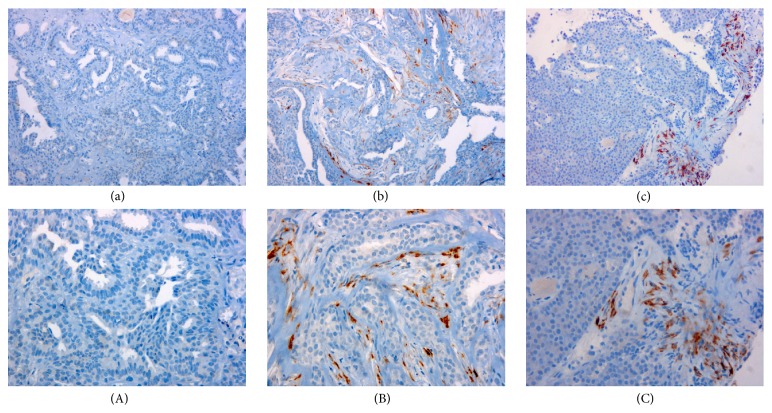
Pro-COL11A1 expression in breast papillary lesions. Immunostaining for pro-COL11A1 in: (a) benign intraductal papilloma, (b) malignant relapse intraductal papilloma, (c) encapsulated papillary carcinoma. Counterstain with Hematoxilin. Lowercase letters images magnification ×200, uppercase letters images magnification ×400.

Among papillomas with malignant relapse 91% showed positive staining (Figure 1(b)), whereas those papillomas with benign or not recurrence present only 33% of immunostaining (*P* < 0.0001) (Figure 1(a)). All five encapsulated papillary carcinoma were positive for COL11A1 staining (Figure 1(C)).

Pro-COL11A1 staining showed sensitivity of 91% and a specificity of 67% when compared intraductal papilloma malignant relapsed samples with those not recurrent. Moreover, Cox regression analysis for recurrence risk presents highly statistical significance (*P* = 0.0008) while comparing positive and negative staining, with a HR of 12.6 (3.8–41.4) (Figure 1 sup data) (see Supplementary Material available online at http://dx.doi.org/10.1155/2015/812027).

## 4. Discussion

The present work demonstrates that the presence of COL11A1 in the stroma of breast intraductal papillomas could be a potential marker of malignant behavior.

Breast intraductal papillomas are considered as benign indolent lesions but a significant number of patients are suitable to develop a malignant recurrence [26] something that explain the huge controversy over the treatment to be applied in these kind of lesions [4, 27–30].

Several clinical groups argue for an aggressive complete excisional treatment when an intraductal papilloma is diagnosed, going from a tumorectomy whether solitary papillomas to a radical mastectomy in the case of diffuse lesions [11, 12, 31–33]. On the other hand there are works suggesting the treatment of breast intraductal papilloma to be not so invasive and based in a conservative image-controlled follow-up [13–15, 34]. The possibility of making a recommendation for excision only in specific cases where an uncertain degree of malignancy is present is also discussed [35]. This could be a nice approach but, how is the malignancy probability of a pure intraductal papilloma determined [8]? The answer must be coming from morphology and characteristics of neoplastic as well as stromal cells.

Breast intraductal papilloma presents a high rate of underestimation (12–19%) when it is diagnosed in Core-Needle Biopsy [10, 29, 36] mainly due to small sample and to indefinite histopathological features [29]. This is why a reliable system for classifying papillary lesions according to malignant potential is required.

Although several biomarkers have been suggested for differentiating potential malignant phenotype of benign intraductal papillomas, none has been demonstrated as an accurate predictive factor of malignancy. Markers as the CD44 [37] or cyclin D1 [38] have been proposed as differentially expressed genes between malignant and benign papillary lesions, but there is no correlation with malignant recurrence of intraductal papillomas. Some genetic alterations, such as loss of heterozygosity of chromosome 16 [39], have also been proposed as capable of predicting an increased susceptibility for malignant recurrence of intraductal papillomas, but accuracy has not been demonstrated [38, 39].

Our work is in the cutting edge for classification of intraductal papillomas because it is based on tumor associated fibroblasts and not in the neoplastic cells by itself or in the presence or absence of myoepithelial cells. COL11A1 expression in fibroblast surrounding central fibrovascular stalks of intraductal papillomas can predict future malignant relapse with a sensitivity of 91%. Although the specificity derived from our data is not so high (65%) it can be explained primarily because the elective treatment for intraductal papillomas in Spain is the complete excision of the lesion, which prevents secondary recurrence.

Positive staining in all encapsulated papillary carcinoma suggests what has been discussed for some time that these lesions, long considered variations of DCIS, may in fact be a form of low-grade invasive carcinoma with an expansile growth pattern [40, 41]. This fact supports our hypothesis of a dual nature of intraductal papillomas: malignant papillary carcinomas or intraductal papillomas with benign prognosis.

This marker combined with other prognostic events such as size larger than 1.5 cm, location [28], or presence of microcalcifications [42] can assist when deciding the possibility of an aggressive treatment versus a conservative follow-up. In any case, the absence of COL11A1 in a biopsy can predict with a high probability that an intraductal papilloma will present a benign behavior since it presents a recurrence HR value of 0.0793 (0.02–0.26), although changing in therapeutic behavior seems complicated without further studies.

Given that this injury occurs predominantly in pre- and postmenopausal [30] women and that breast intraductal papillary lesions are usually hormone-dependent [43] (in our series more than 85% estrogens positive), these patients may be susceptible to receive an chemoprevention with hormone inhibitors. It has been demonstrated in different studies that the inhibition of both estrogen receptors (tamoxifen and raloxifene) [44–46] and aromatase pathway (exametasane) [47] reduces contralateral breast cancer relapse. The major problem of these therapies is the election of patients to receive treatment, we propose that COL11A1 positive biopsy should be a new factor to ponder besides a Gail 5-year risk score greater than 1.66% and prior preneoplastic lesion [47] to select candidates for this chemoprevention as these lesions have a high susceptibility to malignant relapse.

To conclude, the expression of COL11A1 in breast intraductal papillomas is an optimal prognostic biomarker, and we propose that patients with positive staining for this protein should be given further evaluation of both surgical treatment and preventive adjuvant chemotherapy.

## Supplementary Material

SD Figure1: Kaplan-Meier recurrence free analysis across categories of COL11A1. Cox regression analysis presents highly statistical significance (*P*=0.0008) while comparing positive and negative staining, with a HR of malignant recurrence of 12.6 (3.8–41.4) when positive immunostaining for proCOL11A1 appears in the core-needle biopsy of Intraductal Papilloma.
