# Supplementary figures and images for: Collagen Type XI Alpha 1 Expression in Intraductal Papillomas Predicts Malignant Recurrence
Source: Biomed Res Int. 2015 Sep 13;2015:812027. doi: 10.1155/2015/812027 (PMC4584034; doi:10.1155/2015/812027)

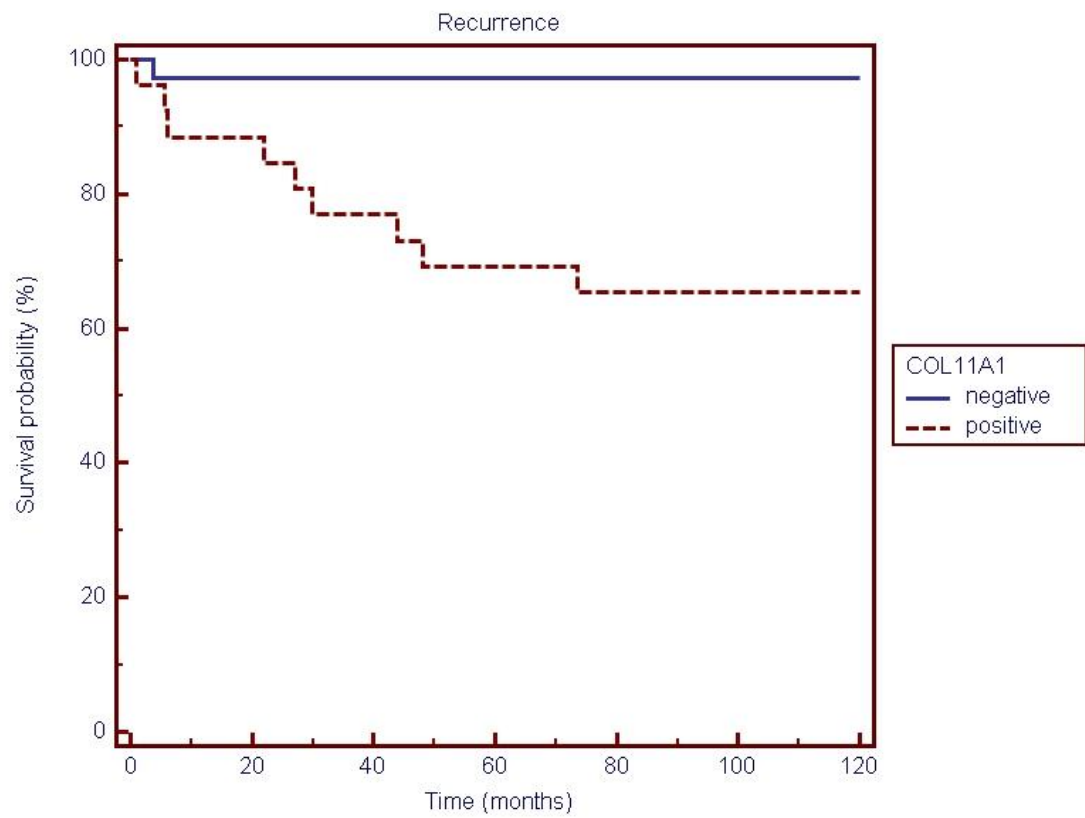

SD Figure 1. Kaplan-Meier recurrence free analysis across categories of COL11A1.

Supplement: Supplementary file 1 — SD Figure1: Kaplan-Meier recurrence free analysis across categories of COL11A1. Cox regression analysis presents highly statistical significance (P=0.0008) while comparing positive and negative staining, with a HR of malignant recurrence of 12.6 (3.8–41.4) when positive immunostaining for proCOL11A1 appears in the core-needle biopsy of Intraductal Papilloma. [file 812027.f1.pdf]
